# Efficacy of Low-Intensity Extracorporeal Shock Wave Treatment in Erectile Dysfunction Following Radical Prostatectomy: A Systematic Review and Meta-Analysis

**DOI:** 10.3390/jcm11102775

**Published:** 2022-05-14

**Authors:** Beom Yong Rho, Si Hyeon Kim, Ji-Kan Ryu, Dong Hyuk Kang, Jong Won Kim, Doo Yong Chung

**Affiliations:** Department of Urology, Inha University School of Medicine, Incheon 22212, Korea; urhobeom@gmail.com (B.Y.R.); housin21@naver.com (S.H.K.); rjk0929@inha.ac.kr (J.-K.R.); dhkang@inha.ac.kr (D.H.K.); urostar@inha.ac.kr (J.W.K.)

**Keywords:** erectile dysfunction, extracorporeal shockwave, penile rehabilitation, radical prostatectomy

## Abstract

Erectile dysfunction (ED) is a well-known complication of radical prostatectomy (RP). Oral 5-phosphodiesterase inhibitors are currently the most widely used penile rehabilitation treatment for ED following RP, but they are less effective than for those with general ED. Low-intensity extracorporeal shock wave treatment (LI-ESWT), causing a biological change that induces neovascularization, has recently been used as a treatment for ED. Therefore, we conducted a systematic review and meta-analysis to investigate the efficiency of LI-ESWT in ED following RP. PubMed, Embase, and the Cochrane Library were searched up until December 2021. The endpoint was the change in IIEF scores after LI-ESWT. Five papers (460 patients) were included in the final analysis. In IIEF scores performed 3–4 months after LI-ESWT, the group receiving LI-ESWT showed statistically significantly better results than the control (WMD = −2.04; 95% CI, −3.72 to −0.35; *p* = 0.02). However, there were a total of two studies that measured the results after 9–12 months. There was no statistical difference between the two groups (WMD = −5.37; 95% CI, −12.42 to 1.69; *p* = 0.14). The results of this analysis indicate that LI-ESWT showed a statistically significant effect on early recovery in penile rehabilitation of ED following RP. However, the level of evidence was low. Therefore, careful interpretation of the results is required.

## 1. Introduction

Erectile dysfunction (ED) is a well-known complication of radical prostatectomy (RP) and radical cystoprostatectomy [1]. The prevalence of ED following RP is reported to be very broad, ranging from 14% to 90%, depending on surgical skill and experience [2]. Advanced surgical techniques and approaches such as robotic surgery are being developed to reduce complications. Nevertheless, there is always varying degrees of nerve damage, such as a cavernous nerve injury, even in nerve-sparing techniques, because of surgical trauma and ischemic damage [3,4]. Some researchers have indicated that, with the help of a penile rehabilitation program, satisfactory sexual function can be restored within 12 to 24 months after surgery [5]. Oral 5-phosphodiesterase inhibitors (PDE5Is) are currently the most widely used penile rehabilitation treatment for ED after RP [6]. However, the response rate to the currently available PDE5Is is much lower in men with ED following RP than in the general ED population [7]. In addition, intracavernous injection therapy using vasodilators can be used, and a penile prosthesis can be inserted if the patient agrees to surgical treatment owing to a poor response to other treatments. A high satisfaction rate has been reported after penile-prosthesis surgery [8,9]. Therefore, functional and structural rearrangements of the damaged penile neurovascular system are necessary to overcome ED after RP [3]. In particular, in the case of low- and intermediate-risk prostate cancer patients who are less likely to receive adjuvant therapy such as androgen deprivation therapy in the future, the results of functional outcomes such as the recovery of ED after surgery have an important effect on the quality of life [10,11].

Low-intensity extracorporeal shock wave treatment (LI-ESWT) has recently been used as a treatment for ED. When LI-ESWT is applied to an organ, the shock wave interacts with the target tissue, causing a biological change that induces neovascularization [12,13]. Therefore, in addition to ED, LI-ESWT is widely used in other fields, such as musculoskeletal disorders, myocardial infarction, and motor neuron damage [14,15,16]. The use of LI-ESWT in overall ED patients has also been reported in recent meta-analyses, with good results [17,18]. However, there are few LI-ESWT studies in patients with ED following RP, and the number of patients included is small [19,20,21,22,23,24]. Therefore, an integrated analysis of these studies is necessary. To investigate the efficacy of LI-ESWT in patients undergoing postoperative penile rehabilitation, we compared the clinical outcomes of LI-ESWT through a systematic review and meta-analysis.

## 2. Materials and Methods

### 2.1. Search Strategy

This study was conducted in accordance with the Preferred Reporting Items for Systematic Reviews and Meta-Analyses (PRISMA) Statement (http://www.prisma-statement.org/) (accessed on 12 May 2022). [25]. A literature search of all publications up until December 2021 was conducted using the Ovid-Embase, PubMed, and Cochrane Library databases. In addition, a cross-reference search of eligible articles was performed to identify studies that were not found in the computerized search. We used combinations of the following MeSH terms and keywords: “prostatectomy”, “shock wave”, “shockwave”, and relevant variants. The search included relevant articles. Two authors (S.H.K. and B.Y.R.) independently reviewed the titles and abstracts according to the inclusion criteria. Afterwards, they performed a full-text evaluation of the identified papers. Any disagreement regarding the inclusion of an article was discussed with the third author (D.Y.C.). We included search strategies for the systematic review in Supplementary File S1.

### 2.2. Inclusion Criteria and Study Eligibility

The eligibility of each study was assessed by considering the participants, interventions, comparators, outcomes, and study design approach [26].

(1) Participants: Patients who underwent RP or radical cystoprostatectomy and had normal sexual function before surgery.
(2) Interventions: Patients who underwent LI-ESWT for penile rehabilitation after the operation.
(3) Comparators: Patients who did not receive LI-ESWT for penile rehabilitation after the operation.
(4) Outcomes: Follow-up result of questionnaires that can evaluate erectile function (for example: International Index of Erectile Function (IIEF-5), Expanded Prostate Cancer Index Composite (EPIC), and Erection Hardness Score (EHS)).
(5) Study design: No restriction on the study design so that both randomized controlled trials (RCTs) and observational studies could be included in the analysis.

In addition, the exclusion criteria were as follows: (1) non-human studies; (2) documents not written in English; (3) case series or reports, reviews, guidelines, and editorial comment; and (4) conference abstracts.

### 2.3. Study Quality Assessments

Quality assessments were conducted independently by two reviewers (B.Y.R. and D.Y.C.) and divided into RCTs and non-RCTs. The Cochrane Bias Risk Tool for Quality Assessment, recommended by the Cochrane Handbook for Systematic Reviews of Interventions, was used for the RCTs [27]. It includes the following risk areas for bias: (1) random sequence generation, (2) allocation concealment, (3) blinding of participants and personnel, (4) blinding of outcome assessment, (5) incomplete outcome data, (6) selective reporting, and (7) other potential biases. Each item was evaluated in the following three categories based on the risk of bias: high, low, and unknown. The Newcastle−Ottawa scale was used for the non-RCTs [28]. The three major assessment categories are selection, comparability, and exposure. Each piece of research can receive up to nine stars. A study score of 7–9 indicates high quality, 4–6 indicates high risk, and 0–3 indicates very high risk of bias.

We also assessed the quality of the final results using the Grading of Recommendations, Assessments, Developments, and Evaluation System [29]. It consists of domains for evaluation of the methodology, accuracy of results, consistency of results, immediacy, and risk of publication bias. Based on these criteria, the quality of the evidence was rated as one of four levels (high, moderate, low, and very low).

### 2.4. Statistical Analysis

The weighted mean differences (WMDs) and 95% confidence intervals (CIs) were calculated for continuous variables using the IIEF-5 questionnaire. Heterogeneity was assessed using the Chi-square and I^2^ tests. A Cochran Q statistic *p*-value <0.05 or an I^2^ statistic >50% was used to indicate statistically significant heterogeneity between studies [30]. Based on the degree of heterogeneity, a random-effects or fixed-effects model was applied to calculate the summary measures [31]. The meta-analysis was conducted using Review Manager Version 5.3 (RevMan, Copenhagen: The Nordic Cochrane Center, The Cochrane Collaboration, Copenhagen, Denmark, 2013). Statistical significance was set at *p* < 0.05 [32]. For the analysis of less than 10 studies, no funnel plots were used to assess publication bias [33].

## 3. Results

### 3.1. Systematic Review Process

**Figure 1:**
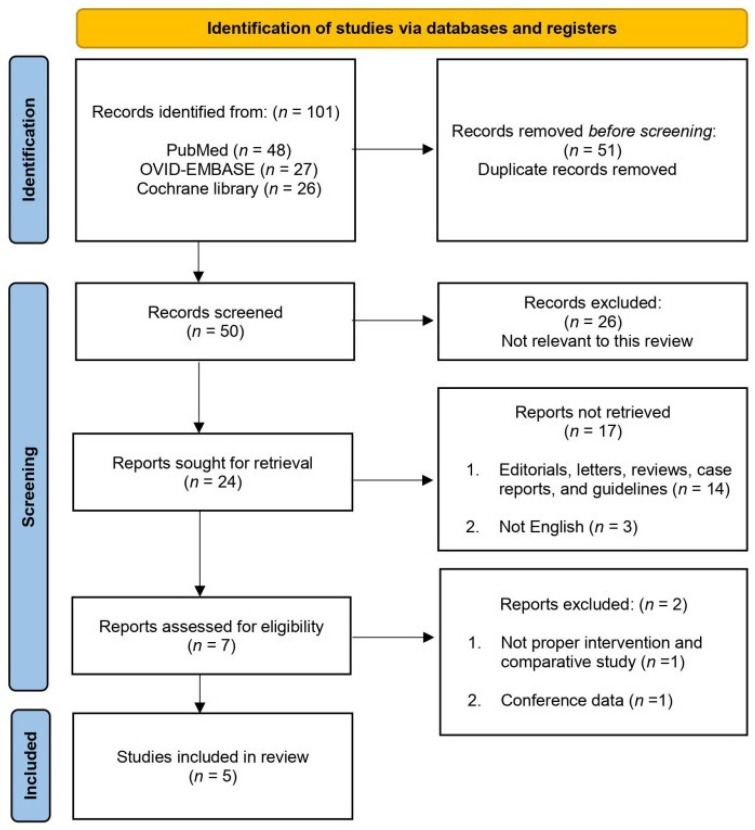
Study selection flowchart according to the Preferred Reporting Items for Systematic Reviews and Meta-Analysis Guidelines.

The PRISMA guidelines were followed, and a flowchart of the study selection process is shown in Figure 1. The initial international database search identified 101 studies (48 from PubMed, 27 from OVID-EMBASE, and 26 from the Cochrane Library), of which 50 remained after the removal of duplicates. After screening the titles and abstracts, 43 articles were excluded. Subsequently, seven full-text articles were evaluated based on pre-established inclusion criteria. As a result, five papers (460 patients) were included in the final analysis (Table 1).

Three studies were RCTs [19,23,24], whereas the others [21,22] were retrospective case-control studies.

### 3.2. Quality Assessment

The quality assessment results based on the Cochrane risk-of-bias tool are shown in Table 2 [19,23,24]. For ethical reasons, the study by Ladegaard et al. [23] allowed the continued use of other erection aids, including penis rings and penile vacuum pumps, for the duration of the study. It is not known whether the participants and outcomes were blinded in all of the RCT studies. Therefore, it was considered high risk.

The results of the quality assessment using the Newcastle–Ottawa scale for non-RCT studies are shown in Table 2 [21,22]. Two studies received six [21] and seven [22] points, respectively, indicating a high quality. In all non-RCT studies, there were no major problems, except for the selection of the control and non-response rates. However, in the study by Inoue et al. [21], the size difference between the control and experimental groups was too large.

### 3.3. IIEF-5 Questionnaire

There was each only one study using the Expanded Prostate Cancer Index Composite (EPIC) and Erection Hardness Score (EHS) questionnaires, so the change in the IIEF-5 questionnaire was used as an endpoint in the final analysis. The endpoint was the change in the IIEF score, which was used to evaluate erectile function.

Statistical analyses using only the RCT study and statistical analyses using all of the studies were performed.

#### 3.3.1. A. RCT Studies 

A total of 200 patients were included in the RCTs. The IIEF scores were analyzed at baseline, after 3–4 months, and after 9–12 months.

**Figure 2:**
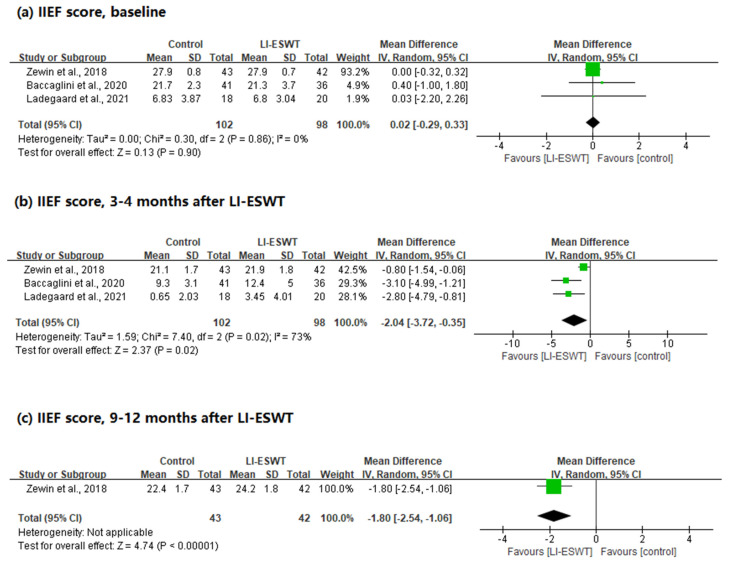
Forest plots for the change in IIEF scores after LI-ESWT (RCT studies).

First, there was no statistical difference in the baseline IIEF scores between the two groups, and no heterogeneity was observed (WMD = 0.02; 95% CI, −0.29 to 0.33; *p* = 0.90; I^2^ = 0%). Next, in the IIEF scores performed 3–4 months after LI-ESWT, the group receiving LI-ESWT showed statistically significantly better IIEF results than the control group, and heterogeneity was observed (WMD = −2.04; 95% CI, −3.72 to −0.35; *p* = 0.02; I^2^ = 73%). Finally, only one study measured the outcome after 9–12 months (WMD = −1.80; 95% CI, −2.54 to −1.06) (Figure 2).

#### 3.3.2. B. RCT and Non-RCT Studies 

In this analysis, 266 patients were included in a total of four studies, with three RCTs and one non-RCT. This was analyzed in the same manner as described above.

**Figure 3:**
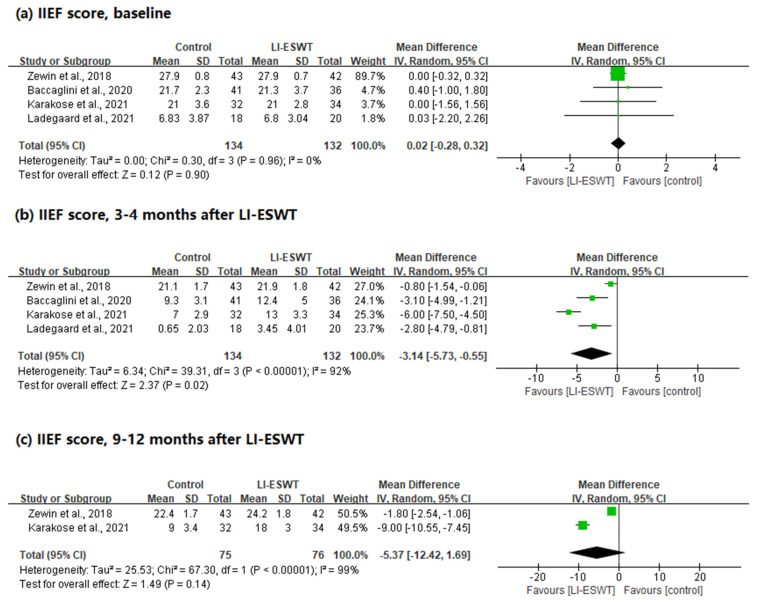
Forest plots for the change in IIEF scores after LI-ESWT (total studies).

There was no statistical difference in the baseline IIEF scores between the two groups, and no heterogeneity was observed (WMD = 0.02; 95% CI, −0.28 to 0.32; *p* = 0.90; I^2^ = 0%). In the IIEF scores performed 3–4 months after LI-ESWT, the group receiving LI-ESWT showed statistically significantly better scores than the control group, and heterogeneity was observed (WMD = −3.14; 95% CI, −5.73 to −0.55; *p* = 0.02; I^2^ = 92%). Finally, there were two studies that measured the results after 9–12 months. There was no statistical difference between the two groups, and heterogeneity was observed (WMD = −5.37; 95% CI, −12.42 to 1.69; *p* = 0.14; I^2^ = 99%) (Figure 3).

### 3.4. The Quality of Evidence Using the GRADE Approach

The assessment of the quality of evidence of each comparison using the GRADE approach is shown in Table 3.

## 4. Discussion

Regarding the biological effects of LI-ESWT, the focus was mainly on angiogenesis and local neovascularization. It was shown that in vitro and in vivo LI-ESWT enhanced the expression of the vascular endothelial growth factor [34,35,36]. Focusing on this, Vardi et al. [37] reported on the use of LI-ESWT in patients with ED. They treated 20 patients twice a week for 3 weeks, which was repeated after a rest period of 3 weeks. Patients with vasculogenic ED were the participants, and the efficiency of LI-ESWT showed a significant increase in IIEF after 1 month and good results were maintained even after 6 months. After this study was published, several studies on LI-ESWT were published, and some conflicting results have been reported. For example, Yee et al. [38] conducted an experiment with settings similar to those of the study protocol of Vardi et al. [37]. The examination of IIEF-5 and EHS scores after 13 weeks in a total of 58 patients (29 patients each) revealed no statistically significant differences. However, meta-analyses of RCTs on LI-ESWT for ED treatment have been published. A total of 833 patients from 14 RCTs were included in the meta-analysis published by Lu et al. [17], which showed that patients who underwent LI-ESWT had significantly improved IIEF (WMD: 2.00; 95% CI, 0.99–3.00; *p* < 0.0001) and EHS (risk difference: 0.16; 95% CI, 0.04–0.29; *p* = 0.01) scores compared with the control. Subsequent studies have reported similar results [18,39]. However, most of the patient groups in these studies were patients with vasculogenic ED and Peyronie’s disease. Therefore, these results are insufficient to explain the effect of LI-ESWT on ED following RP.

The first study to report the effect of LI-ESWT on ED following RP was published by Frey et al. [20]. They conducted a pilot study examining the effects of LI-ESWT on 18 bilaterally nerve-sparing RP patients. A study without a control group reported that LI-ESWT was effective in patients with ED after RP. In addition, in an experimental study of a rat model of pelvic neurovascular injuries, it was reported that LI-ESWT may support nerve recovery and regeneration by directly stimulating neuronal proliferation or indirectly via activation of the supporting functions, such as Schwann cells and angiogenesis [40]. Since then, recent LI-ESWT studies on penile rehabilitation after RP or cystoprostatectomy have been published. However, as mentioned earlier, such studies have not yet been conducted in large-scale RCTs. Therefore, we performed a meta-analysis of published studies to obtain better evidence. A total of five studies were searched for this topic; however, Inoue et al. evaluated ED using EPIC instead of IIEF, unlike the other studies. Therefore, it was difficult to include it in this analysis [21]. Ladegaard et al. showed the results as the amount of change in IIEF, but it did not significantly affect the analysis of the results; therefore, it was included in our analysis [23]. Our study results showed that the ED recovery rate in the LI-ESWT group was significantly higher than that in the control group 3–4 months after LI-ESWT. However, in the 9–12-month long-term results, there were low numbers in the study, and the results were not statistically significant in all of the studies. The exact mechanism of LI-ESWT in ED following RP remains unknown. In summary, it is thought that stimulation by shockwave microbubbles causes neoangiogenesis by activating vascular endothelial growth factor release and endothelial progenitor cells, which also causes stem cell recruitment and Schwann cell activation, leading to nerve regeneration [40,41,42,43]. A study using a mouse model of cavernous nerve injury reported similar results. We were able to observe the recovery of not only vascular regeneration factors, but also various nerve regeneration factors, such as nerve growth factor (NGF), brain-derived neurotrophic factor, and neurotrophin-3, in the experiment of administering an antibody of proNGF associated with microvascular dysfunction [3]. However, these new drugs, including neuromodulation research in ED, are still at the preclinical level [44,45]. On the other hand, we think the current results are more meaningful because LI-ESWT is at a level that can be currently applied in clinical practice. However, our study had several limitations. First, the protocol for performing LI-ESWT in each study was different, as was the use of PDE5Is. This is because there is still no well-established protocol for LI-ESWT in ED, and each study was designed for each situation, focusing on previous study protocols. The clinical results of LI-ESWT were closely related to the energy flux density (EFD). Most of the studies [19,21,22,24] included in this study used an EFD of 0.09 mJ/mm^2^, except for the study by Ladegaard et al. [23] (0.15 mJ/mm^2^). However, in other studies regarding LI-ESWT related to ED, the range of 0.09 to 0.25 mJ/mm^2^ has varied [46,47]. The best EFD for ED treatment has not yet been established. When looking at the use of organs other than the penis for ED, the EFD was set differently depending on the situation. For example, in a study to accelerate angiogenesis in skin burns, 0.04 mJ/mm^2^ was used [48], and studies showing that it is effective for musculoskeletal disorders have reported that it can be increased to 0.3 mJ/mm^2^ [49]. In the current ED study, 0.09 mJ/mm^2^, which was first reported by Vardi et al., was the most used [37], but additional research is still needed. Second, the number of included studies and patients may have been inadequate to provide sufficient evidence. Therefore, caution is needed when interpreting the results of this meta-analysis because the evidence is low. Despite these limitations, our study is valuable as the first meta-analysis of LI-ESWT in ED following RP. In ED following RP, there is currently no specific treatment other than the use of PDE5Is. Although the long-term efficiency and precision protocol are still unclear, our results suggest that LI-ESWT should be considered by clinicians for penile rehabilitation in ED following RP. In addition, we believe that our study statistically demonstrated the effectiveness of LI-ESWT for the early recovery of ED after RP, which is considered a prerequisite for large-scale RCTs.

## 5. Conclusions

In this meta-analysis, LI-ESWT showed a statistically significant effect on early recovery in penile rehabilitation of ED following RP or radical cystoprostatectomy. However, there was no significant difference in the long-term follow-up results, and the data were still insufficient. Therefore, we suggest that LI-ESWT could be an option for early ED recovery after RP. However, the level of evidence was low. Therefore, careful interpretation of the results is required, and additional well-designed large-scale RCT studies are needed.

## Figures and Tables

**Table 1:** Characteristics of the eligible studies.

Authors Year Country	Study Design	Study Summary	Total Patients		Setup of LI-ESWT			Protocol of LI-ESWT Treatment			Follow-Up (months)	Evaluation Tools for ED
					Higher Energy Flux Density (mJ/mm^2^)	Total Pulses /Each Treatment	Pulses /Each Region	No. of Treatments Each Week	No. of Sites	Total Course of Treatment (Weeks)		
Zewin et al. 2018 Egypt	Randomized Clinical Trial	Comparison of penile rehabilitation with or without LI-ESWT after cystoprostatectomy (PDE5Is not used concurrently)	Control	43	0.09	1500	300	2	5	6	1, 3, 6, 9	IIEF EHS
			LI-ESWT	42								
Baccaglini et al. 2020 Brazil	Randomized Clinical Trial	Comparison of penile rehabilitation with or without LI-ESWT after prostatectomy (PDE5Is used concurrently)	Control	41	0.09	2400	600	2	4	8	4	IIEF-5
			LI-ESWT	36								
Inoue et al. 2020 Japan	Non-Randomized Clinical Trial	Comparison of penile rehabilitation with or without LI-ESWT after prostatectomy (PDE5Is used concurrently)	Control	16	0.09	1500	300	1	5	6	3, 6, 9, 12	EPIC
			LI-ESWT	178								
Karakose et al. 2021 Turkey	Non-Randomized Clinical Trial	Comparison of penile rehabilitation with or without LI-ESWT after prostatectomy (PDE5Is used concurrently)	Control	32	0.09	1500	300	2	5	6	3, 6, 12	IIEF-5
			LI-ESWT	34								
Ladegaard et al. 2021 Denmark	Randomized Clinical Trial	Comparison of penile rehabilitation with or without LI-ESWT after prostatectomy (PDE5Is used concurrently)	Control	18	0.15	4000	500	1	6 (twice of each of the penile crurae)	5	1, 3	IIEF-5 EHS
			LI-ESWT	20								

ED, erectile dysfunction; EHS, Erection Hardness Score; EPIC, Expanded Prostate Cancer Index Composite; IIEF, International Index of Erectile Function; LI-ESWT, low intensity extracorporeal shock wave therapy; No., number; PDE5Is, phosphodiesterase-5 inhibitors.

**Table 2:** The results of quality assessment using the Cochrane risk-of-bias tool and Newcastle–Ottawa scale.

A. Results of Quality Assessment of Randomized Control Trial Study by the Cochrane Risk-of-Bias Tool									
Author(s) (Year)	Random Sequence Generation (Selection Bias)		Allocation Concealment (Selection Bias)	Blinding of Participants and Personnel (Performance Bias)		Blinding of Outcome Assessment (Detection Bias)	Incomplete Outcome Data Addressed (Attrition Bias)	Selective Reporting (Reporting Bias)	Other Bias
Zewin et al. (2018) [24]	Low risk		Low risk	High risk		High risk	Low risk	Low risk	Unclear
Baccaglini et al. (2020) [19]	Low risk		Low risk	High risk		High risk	Low risk	Low risk	Unclear
Ladegaard et al. (2020) [23]	Low risk		Low risk	High risk		High risk	Low risk	Low risk	Unclear
**B. Results of Quality Assessment of Nonrandomized Studies by the Newcastle–Ottawa Scale**									
**Author(s)** **(Year)**	**Selection (4)**				**Comparability (2)**	**Exposure (3)**			**Total Score**
	**Adequate Definition** **of Cases**	**Representativeness** **of Cases**	**Selection of Controls**	**Definition of Controls**	**Control for** **Important Factor** **or Additional** **Factor**	**Ascertainment of Exposure**	**Same Method** **of Ascertainment for Cases and Controls**	**Non-Response Rate**	
Inoue et al. (2020) [21]	1	1	0	1	2	1	1	0	7
Karakose et al. (2021) [22]	1	1	0	1	2	1	1	0	7

**Table 3:** Results of the GRADE quality assessment.

Certainty Assessment							Number of Patients		Effect	Certainty
Number of Studies	Study Design	Risk of Bias	Inconsistency	Indirectness	Imprecision	Other Considerations	Control	LI-ESWT	Mean Difference (95% CI)	
IIEF 3–4 Months after LI-ESWT										
3	RCTs	not serious	serious ^a^	not serious	serious ^b^	none	102	98	−2.04 (−3.7, −0.35)	Low
IIEF 9–12 Months after LI-ESWT										
1	RCT	Single study data					43	42	−1.80 (−2.54, −0.35)	
IIEF 3–4 Months after LI-ESWT										
4	RCTs (3) + observational study (1)	not serious	serious ^a^	not serious	serious ^b^	none	212	191	−3.14 (−5.73, −0.55)	Very low
IIEF 9–12 Months after LI-ESWT										
2	RCTs (3) + observational study (1)	not serious	serious ^a^	not serious	serious ^b^	none	212	191	−5.37 (−12.42, −1.69)	Very low

CI, confidence intervals; IIEF, International Index of Erectile Function; LI-ESWT, low intensity extracorporeal shock wave therapy; RCT, randomized clinical trial; ^a^ high I^2^ and clinically relevant; ^b^ total number of participants is small.

## Supplementary Materials

The following are available online at https://www.mdpi.com/article/10.3390/jcm11102775/s1, Supplementart File S1: Search strategies for systematic review.

## Abbreviations

CI	Confidence intervals
EHS	Erection Hardness Score
ED	Erectile dysfunction
EPIC	Expanded Prostate Cancer Index Composite
IIEF	International Index of Erectile Function
LI-ESWT	Low-intensity extracorporeal shock wave treatment
NGF	Nerve growth factor
PDE5Is	Oral 5-phosphodiesterase inhibitors
RP	Radical prostatectomy
RCT	Randomized controlled trials
WMD	Weighted mean differences
