# Supplementary material for: Efficacy of Low-Intensity Extracorporeal Shock Wave Treatment in Erectile Dysfunction Following Radical Prostatectomy: A Systematic Review and Meta-Analysis
Source: J Clin Med. 2022 May 14;11(10):2775. doi: 10.3390/jcm11102775 (PMC9145026; doi:10.3390/jcm11102775)
Supplement: Supplementary file 1 [file jcm-11-02775-s001.zip › jcm-1667585-supplementary.pdf]

1. **PubMed** (No. of article : 48)

("prostatectomy"[MeSH Terms] OR "prostatectomy"[All Fields] OR "prostatectomies"[All Fields]) AND ((("shock"[MeSH Terms] OR "shock"[All Fields] OR "shocked"[All Fields] OR "shocking"[All Fields] OR "shocks"[All Fields]) AND "wave"[All Fields]) OR ("shockwave"[All Fields] OR "shockwaves"[All Fields]) OR "ESWT"[All Fields])

2. **OVID-EMBASE**

**OVID-EMBASE**		
No.	Search term	No. of article
#1	prostatectomy.m_titl.	26317
#2	shock.m_titl.	78054
#3	wave.m_titl.	45982
#4	shockwave.m_titl.	2197
#5	shock wave.m_titl.	6069
#6	eswt.m_titl.	180
#5	#1 and #2	10
#6	#1 and #3	11
#7	#1 and #4	3
#8	#1 and #5	10
#9	#1 and #6	4
#8	#5 or #6 or #7	27

3. **Cochrane library**

**Cochrane library**		
No.	Search term	No. of article
#1	prostatectomy	
#2	shock wave or shockwave or ESWT	
#3	#1 and #2	26
